# Correlation between gallstones and fasting blood glucose to serum high-density lipoprotein cholesterol ratio among American adults

**DOI:** 10.3389/fmed.2025.1528613

**Published:** 2025-01-30

**Authors:** Bo Wu, Huachao Zheng, Caixiang Zhuang, Jiesheng Mao, Luo Yuncheng, Lidong Huang, Min Li, Zhao Feiyang, Sisi Lin, Pengwei Wang, Yiren Hu

**Affiliations:** ^1^Department of General Surgery, The Third Clinical College of Wenzhou Medical University (Wenzhou People’s Hospital), Wenzhou, China; ^2^Department of Neurology, The Third Clinical Medical College of Wenzhou Medical University (Wenzhou People’s Hospital), Wenzhou, China

**Keywords:** gallstones, fasting blood glucose, high-density lipoprotein cholesterol, NHANES, diabetes

## Abstract

**Background:**

Research indicates that the ratio of fasting blood glucose (FBG) to serum high-density lipoprotein cholesterol (HDL-C) (GHR) can accurately predict many diseases. Nevertheless, the relationship between GHR and the risk of gallbladder stones remains unclear. This study investigates the possible relationship between GHR and the incidence of gallbladder stones.

**Methods:**

This research used information gathered from the National Health and Nutrition Examination Survey (NHANES) between March 2017 and March 2020. A calculation was made to determine the GHR by dividing the fasting blood glucose level by the HDL-C level. Several statistical methods, including analysis of threshold effects, smoothed curve fitting, multiple logistic regression modeling, and subgroup analysis, were utilized to investigate the connection between GHR and gallstones.

**Results:**

In 3898 U.S. adults, GHR was significantly positively associated with the prevalence of gallbladder stones. In a fully adjusted model, the incidence of gallbladder stones increased by 7% with each 1-unit increase in GHR (OR [95% Cl] = 1.07 [1.02, 1.14]). Compared with members in the low group, those in the high group had a 100% higher likelihood of getting gallbladder stones (OR [95% CI] = 2.00 [1.31, 3.04]), and this stabilizing connection was always present in the different subgroups. With the help of smooth curve fitting, the research also showed that there was a connection that was formed like an upside-down L shape between GHR and gallbladder stones. The analysis of the threshold effect revealed that the inflection point was 4.28.

**Conclusion:**

The results revealed an inverted L-shaped connection between GHR and gallbladder stones. Keeping GHR levels within a certain range is associated with a lower incidence of gallstones in the general population.

## 1 Introduction

Gallstone disease is the most common disease of the hepatobiliary system and has many common symptoms, including abdominal pain, nausea, abdominal discomfort, vomiting, and poor appetite, which imposes a considerable economic and medical burden on society (1–3). Gallstones are categorized into three types: cholesterol stones, bilirubin stones, and mixed stones, with cholesterol stones and cholesterol-based mixed stones comprising more than 80% of all stones (4). Moreover, gallbladder stones are often accompanied by cholecystitis, which can develop into pancreatitis, bile duct stones, and other acute abdominal conditions, thus posing a great risk to patients’ quality of life and health (5). There are many risk factors for gallstone disease, including advanced age, genetic predisposition, female gender, metabolic syndrome, lifestyle factors, and insulin resistance (IR) (6). Notably, the United States performs over 700,000 cholecystectomies a year at an estimated cost of $6.5 billion (7). Therefore, exploring effective clinical indicators that allow early detection of risk factors for gallstone disease and intervention in gallbladder stone disease is critical for reducing this public health burden (8).

Studies have shown that the development of gallbladder stones is strongly influenced by metabolic disorders, which are strongly correlated with dyslipidemia, hypertension, type 2 diabetes mellitus (T2DM), and obesity (9). Several research investigations have demonstrated the significant effects of serum glucose and IR on the development and progression of gallbladder stones; specifically, studies in high-risk Hispanic populations have shown that IR alters gallbladder function by promoting the production of cholesterol-per-saturated bile, which leads to the formation of gallstones (10). Animal experiments confirm these findings, showing that mice with isolated hepatic insulin resistance exhibit an increased propensity for cholesterol gallstone formation (11). In addition, HDL-C is known as “good cholesterol” and elevated serum HDL-C stimulates the formation of primary bile acids that dissolve cholesterol and reduce the saturation of bile with cholesterol (12). The primary mechanism through which cholesterol is irreversibly eliminated from the body is through bile. Moreover, HDL-C may also be involved in transporting extra cholesterol from lipid-carrying macrophages in the blood vessel wall back to the liver for excretion into bile (13). Owing to the opposite trend between glucose and HDL-C, the difference in GHR is greater between the gallbladder stone group of patients and the non-gallbladder stone group, making it a potential biomarker for the diagnosis of gallbladder stones, providing greater feasibility and cost-effectiveness in clinical applications (14, 15). The primary goal of the current study was to evaluate the connections between them, including the impact of continuous changes in the GHR index, on the basis of the National Health and Nutrition Examination Survey (NHANES), a large dataset. Based on this, the research initially identified the population to focus on. This approach enables easier and more accurate assessment of individual gallstone risk. This study provides empirical support for early screening and individualized diagnostic and treatment strategies for gallstones and helps to develop more targeted clinical interventions.

## 2 Materials and methods

### 2.1 Survey description

The goal of the American research program NHANES is to evaluate the health and nutritional status of both adults and children. One thing that sets it apart from other methods is that it includes both interviews and physical examinations (16). To measure the health and nutritional status of the U.S. population effectively, stratified multistage probability sampling was employed to create a representative sample for the research. The National Center for Health Statistics (NCHS) Research Ethics Review Board approved the study methods. At the time of recruitment, each participant provided written consent (17).

### 2.2 Study population

**FIGURE 1:**
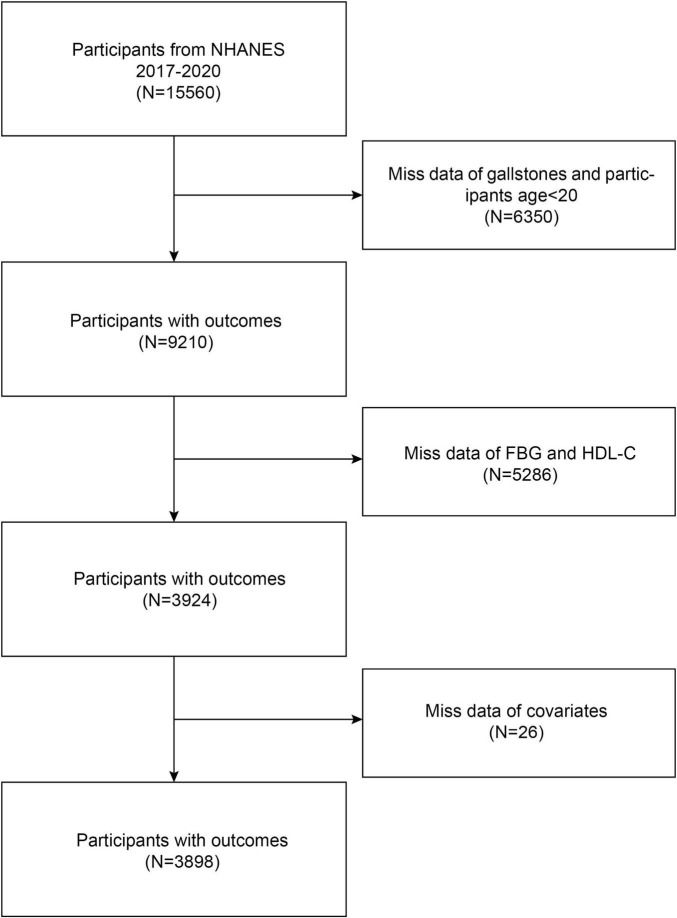
Flow chart of participants’ selection.

Data for this research were gathered from NHANES 2017–2020 March. By using stringent inclusion and exclusion criteria, this study was able to determine the final sample size of 3898 people. The research removed 6350 participants who did not have available gallbladder stone data or refused to answer as well as those who did not know if they suffered from gallstones and were under the age of 20 years, 5286 participants with missing fasting glucose and HDL-C data, and 26 individuals whose covariate values were missing or outliers (Figure 1).

### 2.3 Evaluation of GHR

The biochemical data collected from participants were documented in the NHANES laboratory data section, and the study used the GHR index as an exposure variable in the analyses. The formula for calculating the GHR index was fasting blood glucose (mmol/L)/HDL cholesterol level (mmol/L).

### 2.4 Definition of gallbladder stones

In this study, gallbladder stones were considered an outcome variable. The Medical Condition Questionnaire (MCQ) offered information on the diagnosis of gallbladder stones through self-reported personal interviews, which contained several questions: “Has a doctor or other health professional ever told you that you have gallstones (18)?” If members gave a “yes” answer, they were categorized as members of the group with gallbladder stones. In contrast, members denying this assignment were assigned to the group without gallbladder stones.

### 2.5 Covariates

The correlation between GHR and gallbladder stones was strengthened by adjusting the following covariates (18, 19): Age, gender, race, education, marital status, and the ratio of household income to poverty income (PIR) were included in the demographic data, and the body mass index (BMI) of the normal group was lower than 25 kg/m^2^, while the BMI of the overweight/obese group was greater than or equal to 25 kg/m^2^. Using information from questionnaires, smoking, and alcohol use histories were gathered. Subjects who responded affirmatively indicated that they consumed at least 100 cigarettes during their entire lives, making smoking history a categorical variable. The definition of alcohol consumption status was “Ever had 4/5 drinks or more per day.” As covariates for prior medical history, the research employed diabetes mellitus and hypertension. Data from self-reported personal interviews were used to gather past medical history. There were also laboratory data: such as total cholesterol (TC, mmol/L), total bilirubin (TB, mg/dL), triglyceride levels (TG, mmol/L), and alanine aminotransferase (ALT, U/L) levels. You might receive additional information at http://www.cdc.gov/nhanes.

### 2.6 Statistical analysis

The data analysis in the study was completed with the guidance of the Centers for Disease Control and Prevention (CDC), considering the intricate multi-stage entire cohort survey design and applying the proper NHANES sample weights. The reference group was placed in the quartile with the lowest value (Q1) when the GHR was divided into quartiles. Continuous variables are represented by the mean ± standard deviation (SD), and categorical variables are represented by percentages (%). The *t*-test or chi-square test was used to look for differences between the quartile groupings of the GHR index. Through the utilization of multivariate logistic regression, the linear association that existed between GHR and gallbladder stones was investigated in three distinct models simultaneously. The trend of the linear connection between gallbladder stones and GHR (quartiles) was investigated via a trend test. For interaction and subgroup analyses, three models were used: Model 1 (unadjusted), Model 2 (adjusted only for race, gender, and age), and Model 3 (adjusted for all covariates). Threshold effect analyses and curve-fitting were used in this study to examine any potential nonlinear association between gallbladder stones and GHR. These statistical methods made a more thorough investigation of the possible nonlinear link between GHR and gallbladder stones. Moreover, this study evaluated interaction effects via prespecified effect modifiers. To investigate any variance in these particular groups, stratification variables such as sex, age, education, body mass index, hypertension, smoking, alcohol use, and ALT levels were employed. *P* < 0.05 was selected as the significance criterion for all the statistical analyses, which were conducted via R (version 4.4.2) and EmpowerStats (version 2.0).

## 3 Results

### 3.1 Baseline characteristics of participants

For this study, 3898 competent individuals were obtained, with an average age of 51.00 ± 17.43 years. There were 48.13% males and 51.87% females. Compared with healthy subjects, individuals with gallbladder stones had higher GHR values; compared with those without gallbladder stones, individuals with gallbladder stones were more prone to be female, older, Mexican American, other Hispanic, or non-Hispanic white, individuals with a history of hypertension, diabetes mellitus, higher BMIs, higher triglyceride indices, and higher fasting blood glucose values (all *P* < 0.05) (Table 1).

**TABLE 1:** Basic characteristics of 3898 participants in the 2017–2020 National Health and Nutrition Examination Survey (NHANES).

Characteristics	Total	Non-stone formers	Stone formers	*P*-value
	***N* = 3898**	***N* = 3480**	***N* = 418**	
**Continuous variables, mean ± SD**				
Age (years)	51.00 ± 17.43	50.12 ± 17.45	58.32 ± 15.41	<0.001
BMI (kg/m^2^)	29.83 ± 7.55	29.54 ± 7.33	33.13 ± 8.54	<0.001
PIR	2.61 ± 1.61	2.62 ± 1.62	2.53 ± 1.51	0.488
ALT (U/L)	22.08 ± 19.40	22.19 ± 19.96	21.15 ± 13.90	0.809
TB (mg/dL)	0.49 ± 0.28	0.49 ± 0.28	0.48 ± 0.29	0.762
TC (mmol/L)	183.82 ± 40.99	184.03 ± 40.63	182.06 ± 43.88	0.184
TG (mmol/L)	1.39 ± 1.02	1.37 ± 1.05	1.50 ± 0.75	<0.001
FBG (mmol/L)	6.23 ± 1.87	6.19 ± 1.85	6.59 ± 1.96	<0.001
HDL-C (mmo/L)	1.40 ± 0.41	1.40 ± 0.42	1.36 ± 0.36	0.299
GHR	4.89 ± 2.22	4.84 ± 2.18	5.25 ± 2.45	<0.001
**Categorical variables, %**				
Gender				<0.001
Male	48.13	50.60	27.51	
Female	51.87	49.40	72.49	
Race				<0.001
Mexican American	12.83	12.64	14.35	
Other Hispanic	10.21	9.91	12.68	
Non-Hispanic White	33.68	32.93	39.95	
Non-Hispanic Black	25.35	26.29	17.46	
Other Races	17.93	18.22	15.55	
Education level				0.792
Less than high school	19.37	19.25	20.33	
High school or GED	23.63	23.56	24.17	
Above high school	57.00	57.19	55.50	
Marital status				0.934
Married/Living with a partner	58.80	58.82	58.61	
Living alone	41.20	41.18	41.39	
Alcohol drinking				0.868
YES	13.37	15.59	15.25	
NO	86.63	84.41	84.75	
Smoked at least 100 cigarettes				0.536
Yes	42.84	42.67	44.26	
No	57.16	57.33	55.74	
Hypertension				<0.001
Yes	38.74	36.75	55.26	
No	61.26	63.25	44.74	
Diabetes				<0.001
Yes	15.85%	14.45%	27.51%	
No	81.12%	82.50%	69.62%	
borderline	3.03%	3.05%	2.87%	

BMI, body mass index; PIR, poverty-to-income ratio; ALT, alanine aminotransferase; TB, total bilirubin; TC, total cholesterol; TG, triglycerides; FBG, fasting blood glucose; HDL-C, high-density lipoprotein cholesterol; GHR, fasting blood glucose to serum high-density lipoprotein cholesterol ratio.

### 3.2 Relationship between GHR and gallbladder stones

The conclusions of the multivariate logistic regression analysis regarding the connection between gallbladder stones and GHR are illustrated in Table 2. Multivariate logistic regression analysis demonstrated a considerable positive association between GHR and gallbladder stones in uncorrected Model 1 (OR [95% Cl] = 1.07 [1.03, 1.12]). Even after controlling for age, race, and sex in Model 2, this strong positive connection persisted (OR [95% Cl] = 1.10 [1.05, 1.14]). Despite controlling for all covariates in Model 3, GHR and gallbladder stones were still strongly positively correlated (OR [95% Cl] = 1.07 [1.02, 1.14]); specifically, the frequency of gallbladder stones rose by 7% for every unit increase in GHR. When GHR was transformed into quartiles, this positive connection persisted (all *P* for trend < 0.05). A 1-unit increase in GHR in the highest quartile (Q4) relative to the lowest quartile (Q1) was linked with a 100% increase in the frequency of gallbladder stones in the completely adjusted Model 3 (OR [95% CI] = 2.00 [1.31,3.04]).

**TABLE 2:** Association between GHR and gallstones in logistic regression analysis.

Gallstones	Model 1		Model 2		Model 3	
	**OR [95%Cl]**	***P***	**OR [95%Cl]**	***P***	**OR [95%Cl]**	***P***
**GHR**						
Continuous	1.07[1.03,1.12]	0.0004	1.10 [1.05,1.14]	<0.0001	1.07 [1.02,1.14]	0.0129
Q1(1.09–3.48)	Ref		Ref		Ref	
Q2(3.49–4.42)	1.54[1.14,2.09]	0.0050	1.95[1.43,2.67]	<0.0001	1.85[1.28,2.68]	0.0011
Q3(4.42–5.65)	1.31[0.96,1.79]	0.0901	1.78[1.29,2.47]	0.0005	1.80[1.21,2.68]	0.0036
Q4 ≥ 5.65	1.69[1.26,2.28]	0.0005	2.30[1.68,3.16]	<0.0001	2.00[1.31,3.04]	0.0012
*P* for trend	1.11[1.03,1.19]	0.0036	1.18[1.10,1.28]	<0.0001	1.14[1.04,1.26]	0.0077

OR, odds ratio; 95% CI, 95% confidence interval. Model 1: no covariates were adjusted. Model 2: adjusted for gender, age, and race. Model 3: adjusted for gender, age, race, PIR, education level, marital status, BMI, hypertension, smoking status, drinking, ALT, TG, TC, and TB.

### 3.3 Detection of nonlinear relationships

**FIGURE 2:**
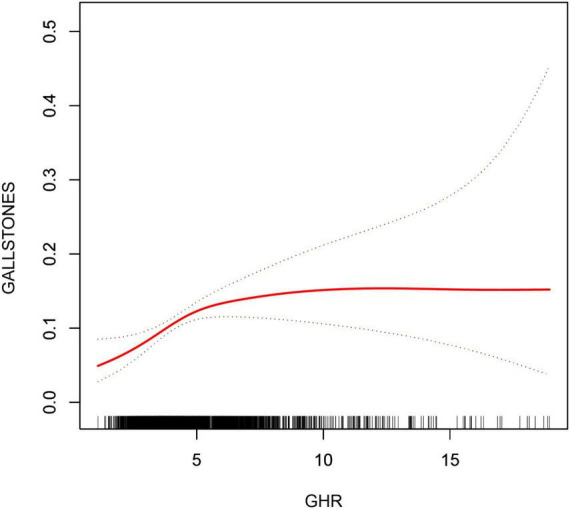
Non-linear associations between GHR and gallstones. The solid red line represents the smooth curve fit between variables. Blue bands represent the 95% confidence interval from the fit.

**FIGURE 3:**
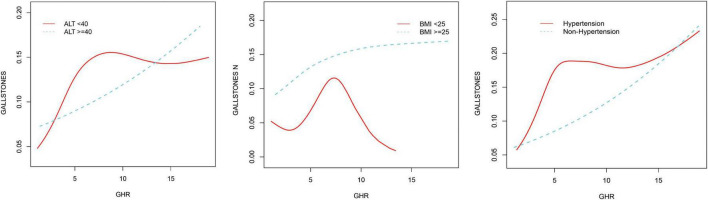
Non-linear associations between GHR and gallstones stratified by ALT, hypertension, and BMI.

By using smoothing curve fitting, this study was able to ascertain the nonlinear saturation relationship between GHR and gallbladder stones, as illustrated in Figure 2. Using threshold effect analysis, the research found that the GHR-gallbladder stone connection had an inflection point of 4.28 (Log-likelihood ratio < 0.001) (Table 3). The inflection point of GHR = 4.28 represents a critical threshold where the effect of GHR on gallstone prevalence plateaus. Below this value, every 1-unit increase in GHR is associated with a 61% increase in gallstone prevalence, while above this threshold, the association becomes statistically insignificant (*P* > 0.05). After stratifying by BMI, ALT, and history of hypertension, the study still observed this saturated nonlinear relationship in the overweight or obese group (BMI = 25), ALT < 40 (U/L), and hypertensive group, with inflection points of 4.14, 4.32, and 4.29 (Log-likelihood ratios of 0.007, <0.001, and 0.002) (Table 4 and Figure 3).

**TABLE 3:** Threshold effect analysis of GHR and gallstones.

Gallstones	OR [95%Cl] *P*
**GHR Total**	
Standard regression model Two-piecewise regression model	0.96 [0.93,0.99] 0.0130
Inflection point	4.28
GHR < Inflection point	1.61 [1.26,2.06] 0.0001
GHR > Inflection point	1.02 [0.95,1.09] 0.5493
*P* for Log-likelihood ratio	< 0.0001

Adjusted for gender, age, race, PIR, education level, marital status, BMI, hypertension, smoking status, drinking, ALT, TG, TC, and TB.

**TABLE 4:** Threshold effect analysis of GHR and gallstones stratified by BMI, ALT, and hypertension.

Gallstones	Adjusted OR (95% CI) *P* value	Adjusted OR (95% CI) *P* value
**GHR**		
Stratified by BMI	<25 (kg/m^2^)	=25 (kg/m^2^)
Standard regression model	1.12 (0.85,1.47) 0.4179	1.05 (0.99,1.11) 0.1306
**Two-piecewise regression model**		
Inflection point	2.92	4.14
GHR < Inflection point	0.37 (0.11,1.30) 0.1211	1.59 (1.15,2.18) 0.0047
GHR **>** Inflection point	1.22 (0.93,1.60) 0.1525	1.01 (0.94,1.08) 0.8071
Log-likelihood ratio	0.098	0.007
Stratified by hypertension	Hypertension	Non-hypertension
Standard regression model	1.06 (0.98,1.13) 0.1339	1.12 (1.01,1.23) 0.0288
**Two-piecewise regression model**		
Inflection point	4.29	3.97
GHR < Inflection point	1.79 (1.24,2.58) 0.0018	1.58 (1.04,2.41) 0.0319
GHR > Inflection point	1.00 (0.92,1.08) 0.9691	1.07 (0.95,1.20) 0.2499
Log-likelihood ratio	0.002	0.084
Stratified by ALT	ALT < 40(U/L)	ALT =40(U/L)
Standard regression model	1.07 (1.00,1.13) 0.0419	1.09 (0.92,1.30) 0.3237
**Two-piecewise regression model**		
Inflection point	4.32	6.05
GHR < Inflection point	1.63 (1.26,2.10) 0.0002	0.88 (0.56,1.37) 0.5673
GHR > Inflection point	1.01 (0.94, 1.09) 0.7948	1.19 (0.94, 1.50) 0.1446
Log-likelihood ratio	<0.001	0.304

### 3.4 Subgroup analysis

To more thoroughly evaluate the strength of the association between GHR and gallbladder stones and to find any possible population variations (Table 5), participants were grouped according to BMI, gender, smoking status, level of hypertension, age, level of alcohol consumption, education level, and ALT level. By subgroup analyses, gallbladder stones and GHR were consistently linked among the groups, including BMI, smoking status, age, level of alcohol consumption, gender, level of education, and ALT level (all *P* for interaction > 0.05). Although the correlation between GHR and gallbladder stones was more pronounced in women, those < 60 years of age, nonsmokers, individuals with less than a high school diploma, those with a drinking habit, non-hypertensive patients, and those with an ALT level of less than 40 (U/L), they did not statistically interact.

**TABLE 5:** The association between gallstones and GHR by selected subgroups.

Subgroup	OR (95% CI) *P*-value	*P* for interaction
Age		0.1109
<60	1.13 (1.04,1.24) 0.0049	
=60	1.03 (0.96,1.11) 0.3986	
Gender		0.2595
Male	1.02 (0.92,1.13) 0.7029	
Female	1.10 (1.02,1.18) 0.0123	
BMI		0.5718
<25 =25	1.13 (0.88,1.47) 0.3422 1.05 (0.99,1.11) 0.1261	
Smoke		0.5243
Yes	1.06 (0.99,1.14) 0.0731	
No	1.10 (1.01,1.20) 0.0323	
Education level		0.4535
Less than high school	1.14 (1.00,1.31) 0.0492	
High school or GED	1.07 (0.96,1.19) 0.1998	
Above high school	1.07 (0.99,1.15) 0.0778	
Drinking		0.0726
Yes	1.20 (1.05,1.38) 0.0080	
No	1.05 (0.99,1.12) 0.1130	
Hypertension		0.5279
Yes	1.06 (0.99,1.13) 0.0787	
No		0.9194
ALT	1.10 (1.00,1.20) 0.0405	
<40	1.07 (1.00,1.14) 0.0354	
=40	1.08 (0.91,1.28) 0.3899	

Gender, age, race, PIR, education level, marital status, BMI, hypertension, smoking status, drinking, ALT, TG, TC, and TB were adjusted. The strata variable was not included in the model when stratifying by itself.

## 4 Discussion

Gallbladder stone incidence and GHR were found to be positively correlated in a recent cross-sectional study with 3898 representative adults. There was no significant dependence on this association in terms of BMI, gender, hypertension, smoking status, age, level of alcohol consumption, level of education, or ALT level in this study. According to these studies, higher GHR levels could be an independent risk factor for gallbladder stones. Additionally, a nonlinear relationship was observed between GHR and gallbladder stones, determining the saturation value of GHR for all participants (4.28). This implies a significant connection between GHR levels and gallbladder stones within a particular range, suggesting that maintaining optimal GHR levels may have clinical value in lowering the incidence of gallbladder stones. The observed inflection point of GHR = 4.28 suggests a threshold beyond which the relationship between GHR and gallstone prevalence weakens. Biologically, this could indicate that GHR levels up to this point significantly reflect metabolic disturbances, such as insulin resistance, dyslipidemia, or impaired glucose metabolism, which are key contributors to gallstone formation. However, once GHR exceeds this threshold, other risk factors, such as obesity, bile composition, and genetic predispositions, may play a more dominant role in gallstone pathogenesis, diminishing the relative contribution of GHR (6). The identification of GHR = 4.28 as an inflection point has important implications for early detection and risk stratification. Maintaining GHR below this threshold could serve as a clinical marker for preventing gallstones in populations at risk of metabolic disorders. Furthermore, this threshold could guide future studies aimed at understanding the underlying mechanisms that contribute to gallstone formation and its interaction with metabolic factors. It is worth noting that above the inflection point, gallstone prevalence may be driven by other factors, emphasizing the multifactorial nature of this condition. Notably, several studies have found that GHR is an indicator of a number of diseases. For instance, a cross-sectional study with over 30,000 members revealed a positive correlation between GHR and the onset of nonalcoholic fatty liver disease (15). Another study explored the connection between acute coronary syndrome and GHR and discovered a high correlation between the two (14). Thus, using information from the NHANES database, the study explored the potential link between GHR and gallstones. Up to now, this is the first research to evaluate the connection between GHR and gallbladder stones. Multiple logistic regression models showed that elevated GHR was independently associated with an increased risk of gallbladder stones. This result facilitates the clinical identification of patients with gallstones. Gallstones were typically diagnosed by imaging tests like abdominal ultrasonography. The GHR was calculated as the ratio of fasting blood glucose and HDL-C levels. Since lipid profiling and complete blood counts are routine tests, GHR data are far more readily available than abdominal ultrasound data. Thus, GHR may be a new and practical way to measure the prevalence of gallstones and assist medical professionals in determining whether a patient needs an abdominal ultrasound in the future. Furthermore, with an inflection point of 4.28, an inverted L-shaped relationship between GHR and gallbladder stones was discovered. Other factors may have an impact on the variation in the relationship between GHR and gallbladder stones on either side of the inflection point. Table 6 demonstrates that patients with GHR ≥ 4.28 had a larger proportion of males, higher proportions of BMI, ALT, triglycerides, diabetes mellitus, hypertension, age level, alcohol intake, and smoking, and lower proportions of PIR and total cholesterol. Abnormalities in these markers are typically related with gallstones (20). When GHR levels surpassed 4.28, a comparatively weak effect of GHR on gallbladder stones was observed, most likely as a result of the existence of additional gallbladder stone risk factors. This study emphasizes that keeping GHR levels within a healthy range is associated with a lower incidence of gallstones in the general population, with a particular emphasis on keeping GHR levels below 4.28; gallbladder stones are much less likely when GHR levels are below the threshold. It is possible to construct new evidence in support of GHR management by utilizing this inflection point.

**TABLE 6:** The baseline characteristics of participants on both sides of the inflection point according to gallstones.

GHR	<4.28	≥4.28	*P*-value
Participants	1822	2076	
Age, year	49.49 ± 17.92	52.33 ± 16.87	<0.001
Male, %	34.96%	59.68%	<0.001
Body mass index, kg/m^2^	27.46 ± 6.56	32.09 ± 7.70	<0.001
HYPERTENSION, %	30.85%	45.66%	<0.001
Diabetes, %	4.94%	25.43%	<0.001
PIR	2.76 ± 1.63	2.49 ± 1.58	<0.001
ALT, U/L	18.97 ± 20.30	24.81 ± 18.16	<0.001
Drinking, %	13.67%	17.21%	0.005
Smoking, %	39.02%	46.19%	<0.001
Total cholesterol, mg/dL	189.81 ± 39.48	178.57 ± 41.57	<0.001
Triglycerides, mmol/L	1.06 ± 0.53	1.67 ± 1.241	<0.001

At present, there could be more than one mechanism involved in the poorly understood underlying mechanisms of this positive association between gallstone occurrence and GHR. First, GHR has been demonstrated to be positively correlated with the onset of nonalcoholic fatty liver disease (15). Today, a variety of metabolic conditions, such as obesity, hypertension, postprandial hyperglycemia, and dyslipidemia, have been indicative of metabolic syndrome (MetS). It is commonly known that increasing obesity rates are linked to higher rates of morbidity and death from a number of the most common diseases in the Western world, such as gallstones (21, 22). Non-alcoholic fatty liver disease (NAFLD) is a manifestation of MetS in the liver and has been discovered to be strongly associated with obesity, hypertension, dyslipidemia, and T2DM (23, 24). Moreover, according to a recent study, gallstone development was highly correlated with NAFLD. It was anticipated that gallbladder stones and NAFLD share a precursor, most likely MetS. Another clear sign of MetS, a collection of cardiometabolic risk factors that include obesity, insulin resistance, diabetes, and hyperlipidemia, was NAFLD (25). Gallstones and metabolic syndrome were revealed to be strongly associated in a recent study, with an OR of 1.42. NAFLD was characterized by insulin resistance, which was a primary cause of MetS because the effects of hyperinsulinemia stimulated anabolic processes and increased the entry of free fatty acids into the liver, which promoted the storage of hepatic fat (1, 26, 27). Additionally, elevated hepatic cholesterol release, increased bile supersaturation, reduced gallbladder motility, and the ease of gallstone formation can all result from hyperinsulinemia (28, 29). Another characteristic of the metabolic syndrome was obesity, which was also associated with gallstones and NAFLD. Numerous problems, including diabetes, fatty liver, and metabolic syndrome, were linked to this. Overproduction of hepatic cholesterol due to increased systemic cholesterol synthesis, fatty acid overload in tissues, and overproduction of hepatic cholesterol led to more lithogenic bile (30). Additionally, a meta-analysis with an RR of 1.5 revealed that diabetes patients had a markedly higher incidence of gallstones, another hallmark of MetS (31). This relationship was also supported by molecular processes. An intestinal hormone called fibroblast growth factor 19 (FGF19) controls several processes, such as metabolic rate, gallbladder filling, and bile acid equilibrium. Patients with gallstones developed biliary stones as a result of insufficient increase in bile acid production by FGF19. Elevated insulin resistance, non-alcoholic fatty liver disease, and diabetes mellitus were linked to altered plasma FGF19 levels (32). Second, aberrant lipid levels have been associated with the development of gallbladder stones; a prior study found that older NAFLD patients were more likely to develop gallstones if their HDL-C levels were lower (33). Serum HDL-C levels and the incidence of gallbladder stones in a European population were found to be negatively linearly correlated in a recent study. This result could be explained by the ability of HDL-C to reduce inflammation and encourage the removal of cholesterol from peripheral tissues (34). Controlling the lipid profile could be a crucial preventive measure in light of recent findings that lipid metabolism played an essential part in the development and formation of gallstones (35).

This study has certain advantages. First, since the study was based on a sizable sample of NHANES data—collected via the stratified multi-stage probability sampling method that underwent stringent quality control—it is more dependable and representative. Second, to minimize the impact of bias on the study, confounding variables were corrected. Third, to ascertain whether the relationship between GHR and gallstone prevalence varied by demographic characteristics, subgroup analyses were conducted. However, this study had some limitations. Firstly, because gallstone data were available, this analysis covered data from only 2017 through March 2020. Secondly, although we adjusted for several known covariates, unmeasured or unknown confounding factors may have influenced the observed associations. These factors could include dietary habits, physical activity levels, or genetic predispositions, which were not accounted for in our analysis. Third, in this study, gallstone diagnosis relied on self-reported data from participants rather than confirmation through imaging techniques or medical records. This reliance on self-reporting introduces a potential for information bias, such as recall bias or inaccurate reporting, which could affect the validity of our results. Self-reported data may underestimate or overestimate the actual prevalence of gallstones, particularly in asymptomatic cases where individuals may not be aware of their condition. This limitation could have a significant impact on our findings. For instance, the reliance on self-reported data may lead to underreporting of asymptomatic gallstones, potentially resulting in an underestimation of the overall gallstone prevalence. Additionally, misclassification due to inaccurate recall or reporting may dilute the observed association between GHR and gallstones. Future studies utilizing objective diagnostic methods, such as imaging or medical record confirmation, would be better suited to provide a more accurate assessment. Furthermore, the cross-sectional nature of this study limits the ability to establish causal relationships between GHR levels and gallstone prevalence. Our findings are restricted to associations, and further longitudinal or interventional studies are required to confirm causality.

## 5 Conclusion

In conclusion, a large sample of American adults revealed that increased GHR was independently linked to an increased incidence of gallbladder stones. In addition, the study identified an inverted L-shaped connection between GHR and gallbladder stones via the smooth curve fitting, with an inflection point of 4.28. This finding implies that a lower prevalence of gallstones in the general population is linked to maintaining GHR levels within a healthy range. Greater numbers of cohort studies or randomized controlled trials are desperately needed in the future to validate these results and offer greater proof.

## Data Availability

The original contributions presented in the study are included in the article/supplementary material, further inquiries can be directed to the corresponding author.
